# Highly Efficient Colored Perovskite Solar Cells Integrated with Ultrathin Subwavelength Plasmonic Nanoresonators

**DOI:** 10.1038/s41598-017-10937-3

**Published:** 2017-09-06

**Authors:** Kyu-Tae Lee, Ji-Yun Jang, Jing Zhang, Sung-Mo Yang, Sanghyuk Park, Hui Joon Park

**Affiliations:** 1 Department of Materials Science and Engineering, University of Illinois at Urbana-Champaign, Urbana, Illinois 61801 United States; 2 Department of Energy Systems Research, Ajou University, Suwon, 16499 Korea; 3 Department of Chemistry, Kongju National University, Chungnam, 32588 Korea; 4 Department of Electrical and Computer Engineering, Ajou University, Suwon, 16499 Korea

## Abstract

Highly efficient colored perovskite solar cells that exploit localized surface plasmon resonances in ultrathin subwavelength plasmonic nanoresonators are demonstrated. Localized resonances in ultrathin metal nano-strip optical resonators consisting of an array of metallic subwavelength nanowires on a transparent substrate, fabricated by using low-cost nanoimprint lithography over a large area, lead to a sharp peak in a reflection spectrum for distinctive color generation with angle-insensitive property up to 60°, and simultaneously transmit the complementary spectrum of visible light that can be efficiently harvested by the perovskite solar cells for electric power generation. The plasmonic color filter-integrated perovskite solar cells provide 10.12%, 8.17% and 7.72% of power conversion efficiencies with capabilities of creating vivid reflective red, green and blue colors. The scheme described in this work could be applied to a variety of applications such as power-generating decorations, tandem cells, power-saving wearable devices and energy-efficient reflective display technologies.

## Introduction

Solar energy is one of the most important and recognized renewable energy sources as it is the cleanest and the most abundant energy resource on earth. With advances in photovoltaic (PV) materials, fabrication processes, device architectures and efficient light management, a power conversion efficiency (PCE) of flat-plate single-junction solar cells and modules closely reach theoretical efficiency limit^1–3^. Organometal trihalide perovskite (PVSK) solar cells in recent years have emerged as a highly appealing PV platform that can provide desired functionalities such as low temperature solution processability, high flexibility, easy scalability, low cost, and comparable performance characteristics with existing inorganic thin-film solar cells^4–11^. There have been numerous attempts to improve the performance of the PVSK solar cells and the PCE continues to increase thus being able to achieve the record efficiency exceeding 20%^12–16^. Despite such great potentials for performance enhancements, the lack of producing vivid color appearances, particularly blue and green colors, of the PVSK solar cells still remains largely challenging so that it is quite difficult for the existing PVSK solar cells to be harmoniously integrated with automotive surfaces and building envelopes such as windows, awnings, walls and facades. Such aesthetical versatility is essential to extensive use of the solar cells for a variety of applications including building-integrated PV (BIPV), self-powered wearable devices, power-saving display systems and power-generating windows^17–32^. Nanostructured color-filtering schemes based on conventional Fabry–Pérot cavities and photonic crystals have been successfully incorporated with the PVSK solar cells^33–35^. However, optical microcavity typically involves two metallic layers that have non-negligible absorptions in the visible wavelength range as they require a certain film thickness to provide reasonably high reflections for strong optical interference effects, thereby causing the PCE of the colored solar cells to be significantly reduced. In addition to the performance degradation, the optical property of both the microcavity and photonic crystals is highly sensitive with respect to angles of incidence, thus dramatically limiting diverse applications. Furthermore, the spectral reflectance and transmittance of these two photonic cavity-integrated colorful solar cell devices exhibit a relatively broad resonance that contains a wide range of off-resonant wavelength components, which cannot be efficiently harnessed by the PVSK solar cells and degrade the color purity at the same time. Hence, there is a critical need to develop a new and simple scheme to address the aforementioned challenges.

In this work, we present high-performance decorative PVSK solar cells creating easily tunable reflective colors with angle invariant features up to 60° by exploiting localized surface plasmon resonances (LSPRs) in an array of ultrathin metallic nanowire patterned at the subwavelength scale on a transparent substrate for the first time. The LSPRs lead to a fairly sharp peak in the reflection spectrum for color generation with high purity and angle-insensitivity. Moreover, the ultrathin thickness of the single metallic layer in the plasmonic subwavelength nanoresonators yields almost negligible absorption in the visible wavelength regime, thereby allowing most of incident light to be efficiently scavenged by the PVSK solar cells for electricity generation. Consequently, highly efficient colored perovskite solar cells having 10.12, 8.17 and 7.72% of the PCE for the red, green, and blue (RGB) colors, respectively, were demonstrated. The approach presented in this work could open the door to a multitude of novel applications including BIPV, power-saving display technologies, tandem solar cells and colored solar panels.

## Results and Discussion

**Figure 1:**
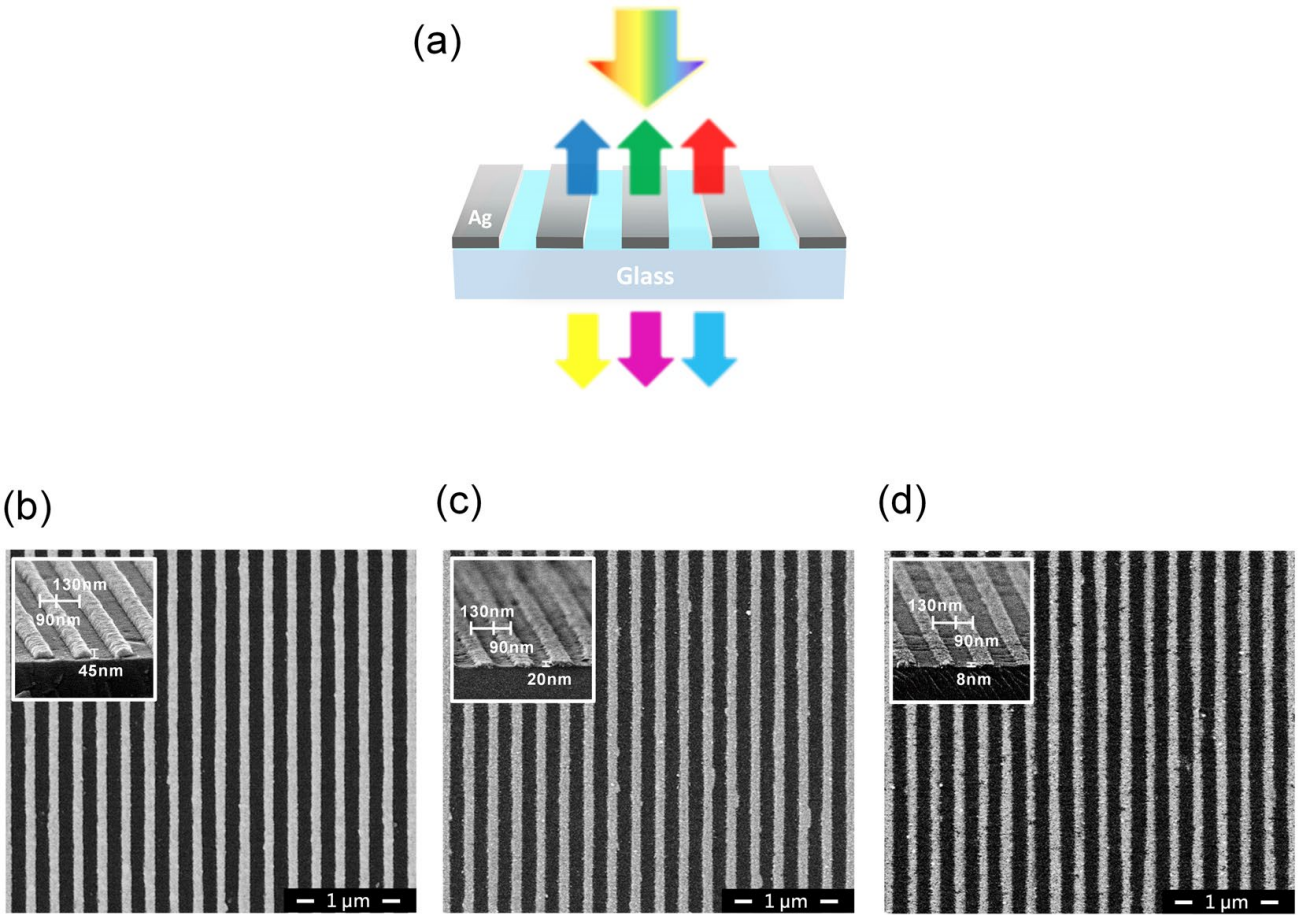
(**a**) Schematic diagram of the ultrathin metallic nanowire-based plasmonic color filters that comprise an array of a single Ag layer patterned at the subwavelength scale on a glass substrate. Top and cross-sectional views of scanning electron microscope (SEM) images of (**b**) blue (B) (**c**) green (G) (**d**) red (R) of the fabricated plasmonic color filters. For producing the BGR reflection colors, the thickness (t) of the Ag gratings is varied to be 45, 20 and 8 nm, respectively, while a period (P = 220 nm) and a width (W = 90 nm) are fixed.

Figure 1(a) shows the schematic diagram of the ultrathin plasmonic color filters comprising a single layer of subwavelength metallic nanowire arrays on a glass substrate for LSPR^36–39^. The period and width of metallic nanowires are fixed as 220 nm and 90 nm, respectively. A metallic film thickness is varied to change the reflection and transmission colors: 8, 20 and 45 nm for RGB reflection, and correspondingly cyan, magenta and yellow (CMY) transmission colors, respectively. As silver (Ag) has the lowest optical absorption loss in the visible wavelength range, which is highly desired for achieving the capability of filtering visible light with high efficiency, Ag with the ultrathin thickness was used to minimize the absorption loss in this study. Figure 1(b)–(d) present top and cross-sectional views of scanning electron microscopy (SEM) images of the fabricated plasmonic color filters made by a simple, low-cost and high-throughput nanoimprint lithography^40^, exhibiting that the periodic subwavelength grating structures with high precision and smooth sidewalls are achieved. As can be seen from the SEM images, the dimensions of the plasmonic color filter structures are well-matched with our target parameters.

**Figure 2:**
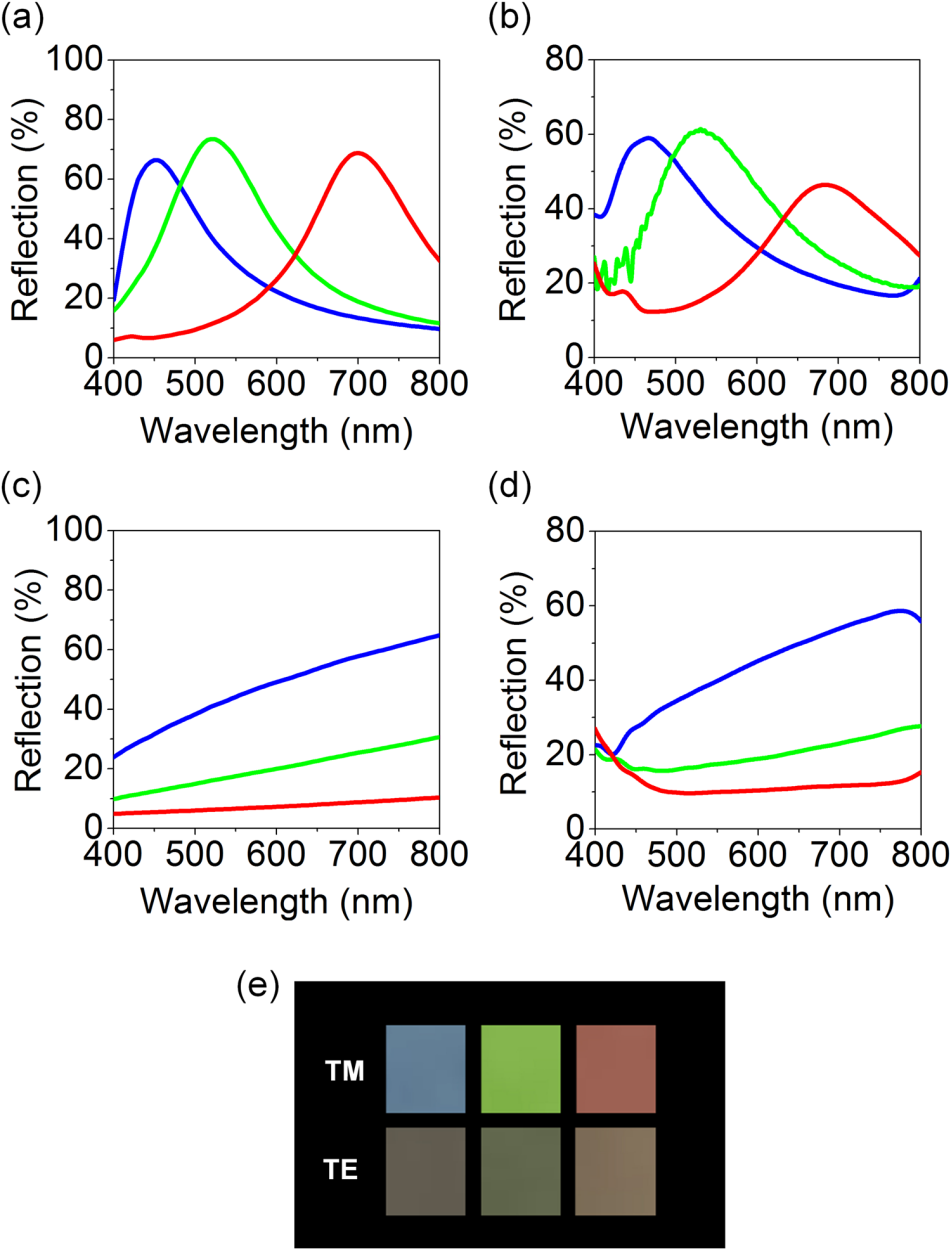
(**a**) Simulated and (**b**) measured spectral reflectance curves at normal incident angle for TM polarization. The simulated spectra show plasmonic resonances at 700, 520 and 450 nm for the RGB colors while the resonances occur at 685, 530 and 465 nm in the measured profiles. (**c**) Simulated and (**d**) measured spectral reflectance curves at normal incidence for TE polarization presenting no resonances in the spectra. (**e**) Optical images of the fabricated plasmonic color filters showing that the RGB reflection colors are observed for TM polarization, while gray colors are noticed for TE polarization.

Simulated spectral reflectance curves of the ultrathin subwavelength plasmonic nanoresonator-based structural colors for transverse magnetic (TM) polarization where an oscillation direction of the electric field of incident light is perpendicular to the direction of subwavelength nanogratings are given in Fig. 2(a), presenting that the simulated reflection spectra show a clear single resonance effect with high efficiency of around 70%. The thicknesses of the Ag layers are 8, 20 and 45 nm for creating the RGB reflection colors, while both the period and width of the subwavelength metallic gratings are fixed at 220 nm and 90 nm. The simulated profiles were obtained by using a commercial COMSOL Multiphysics software that was based on finite element method. The refractive index of Ag, measured by using a spectroscopic ellipsometer (Elli-SE, Ellipso Technology Co.), and 1.46 for the glass substrate were used in the simulation, which are provided in Figure S1. Under the TM-polarized light illumination, the nanostructures strongly reflect a certain component of light in the visible wavelengths due to the LSPR excited in the ultrathin Ag gratings, leading to reflection peaks and thus creating distinctive reflection RGB colors. In a bare Ag film, this extraordinary optical effect, generated by the structures, cannot be found (Figure S2). With increasing the thickness of the Ag gratings, the effective refractive index decreases, thereby moving a resonant wavelength toward the shorter wavelength range^41^. Figure 2(b) describes spectral reflectance curves of the fabricated plasmonic color filters measured by using a spectrometer (Elli-RSc, Ellipso Technology Co.), showing good agreement with the simulated profiles with relatively lower reflection efficiency (~60%). The simulated (measured) profiles show strong plasmonic resonances occurring at 700 (685), 520 (530) and 450 (465) nm for the RGB colors. The small difference of the reflection efficiency and the resonance wavelength between simulation and experimental result could be attributed to fabrication defects, surface roughness, slightly different dimensions and nonparallel light illumination during the measurement. Figure 2(c) and (d) depict simulated and measured reflection spectra at normal incidence under the transverse electric (TE) polarization, where an oscillation direction of the electric field of incident light is parallel to the direction of subwavelength nanogratings, light illumination. For TE polarization, the LSPR cannot be excited, so it is observed that the reflection gets strong with increasing the thickness of the Ag gratings without optical resonance effects in the reflection spectra as can be seen from the figures. Photographs of the fabricated samples taken under the ambient light illumination for both TM (top) and TE (bottom) polarizations are shown in Fig. 2(e), clearly showing that the RGB reflection colors can be seen from the fabricated filters for TM polarization whereas gray colors are observed from the same samples for TE polarization.

**Figure 3:**
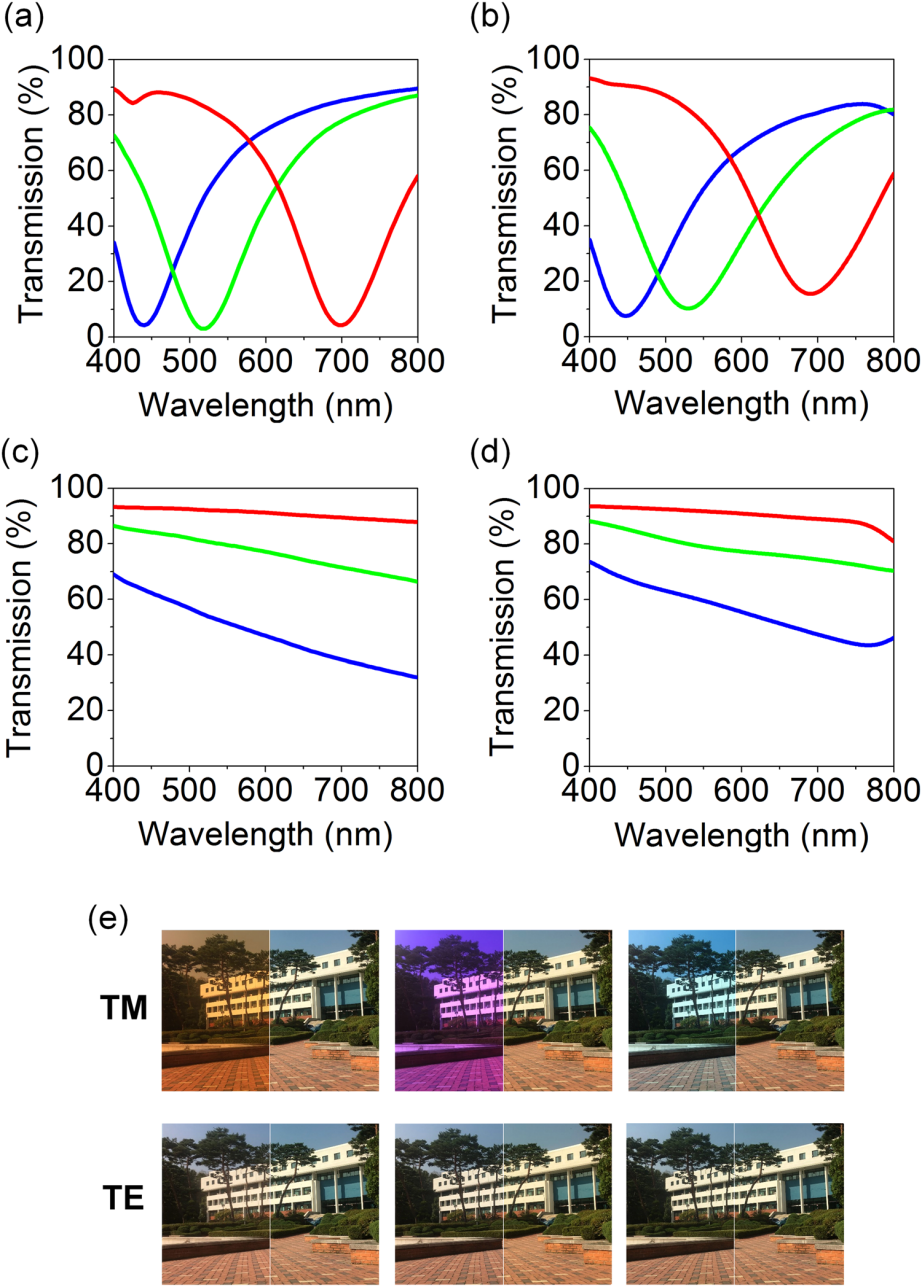
(**a**) Simulated and (**b**) measured spectral transmittance curves at normal incidence for TM polarization exhibiting high brightness and great contrast. The Ag layer that shows the lowest absorptions at visible frequencies with the ultrathin thickness allows a large proportion of visible light to be transmitted with insignificant optical absorption losses. (**c**) Simulated and (**d**) measured spectral transmittance curves at normal incidence for TE polarization. (**e**) Photographs of the fabricated ultrathin nanowire-based plasmonic color filters displaying that the background can be seen through the fabricated devices with the CMY semitransparent colors only for TM polarization.

In Fig. 3(a), simulated transmission spectra of the plasmonic color filters at normal incident angle for TM polarization are described. As the structure that comprises the single Ag layer with the ultrathin thickness results in fairly small absorption, it allows the complementary spectrum to be transmitted through the transparent glass substrate with negligible optical loss, which can be harvested by solar cell for electricity generation to be discussed later. Positions of the reflection peak (i.e., resonance wavelength), shown in Fig. 2(a), are consistent with those of transmission valley, shown in Fig. 3(a). Experimental data measured by using the spectrometer (V-770 UV-Visible-Near Infrared Spectrophotometer, JASCO) are provided in Fig. 3(b), presenting good match with the simulated profiles. The measured spectra have relatively broader profiles as compared to the simulated results, which are ascribed to imperfect fabrications and rough surfaces, causing light scattering. Figure 3(c) and (d) reveal simulated and measured spectral transmittance curves for TE polarization, showing no resonance behaviors. As we observed in the reflection spectra in Fig. 2(c) and (d), no LSPR behaviors are observed due to the polarization of incident light. In Fig. 3(e), optical images of the fabricated plasmonic color filters taken under both TM- and TE-polarized illuminations are displayed, showing that a background building can be clearly seen through the fabricated colored samples with the semitransparent CMY colors featuring high luminance and great homogeneity over a large area (2 cm × 3 cm) for TM polarization (top), while there are no colors for TE polarization (bottom). To evaluate the color purity, color coordinates of the transmission colors are calculated from the simulated and measured spectra and illustrated on the CIE 1931 chromaticity diagram as shown in Figure S3. Such transmitted light with high efficiency can be utilized for electric power generation by integrating solar cells underneath the structural color filters, while producing reflection colors, thereby achieving self-powered reflective display platform and designing colored solar cells, which will be described in the last section. Sunlight that is unpolarized should be considered for solar cell application. Simulated and measured reflection and transmission spectra of the plasmonic color filters at normal incidence under unpolarized light illumination are provided in Figure S4. Due to a flat spectral response for TE polarization, the peaks in the reflection profiles for unpolarized normal incident light are not distinct as compared to those in the reflection spectra for TM polarization. Such broadened resonances for color generation lead to a degraded color purity. On the other hand, the overall transmission efficiency is improved for unpolarized light, which is ascribed to high transmission for TE polarization. It is thus expected that the electrical performance, particularly the current density, is enhanced since the absorption in the PVSK photoactive layer increases.

The colors were tuned by controlling the thickness of Ag nanogratings, however, it is important to note that the color can also be tuned by varying the width (W = 140, 90 and 50 nm for CMY) of the subwavelength gratings at the fixed Ag thickness (*t* = 20 nm) and the period (P = 280 nm) (Figure S5), thus creating colors in a pixel unit via only one lithographic step. This feature is desired for the practical applications, compared with the conventional color-filtering scheme, requiring three separate and accurately aligned lithographic steps to prepare a pixel.

**Figure 4:**
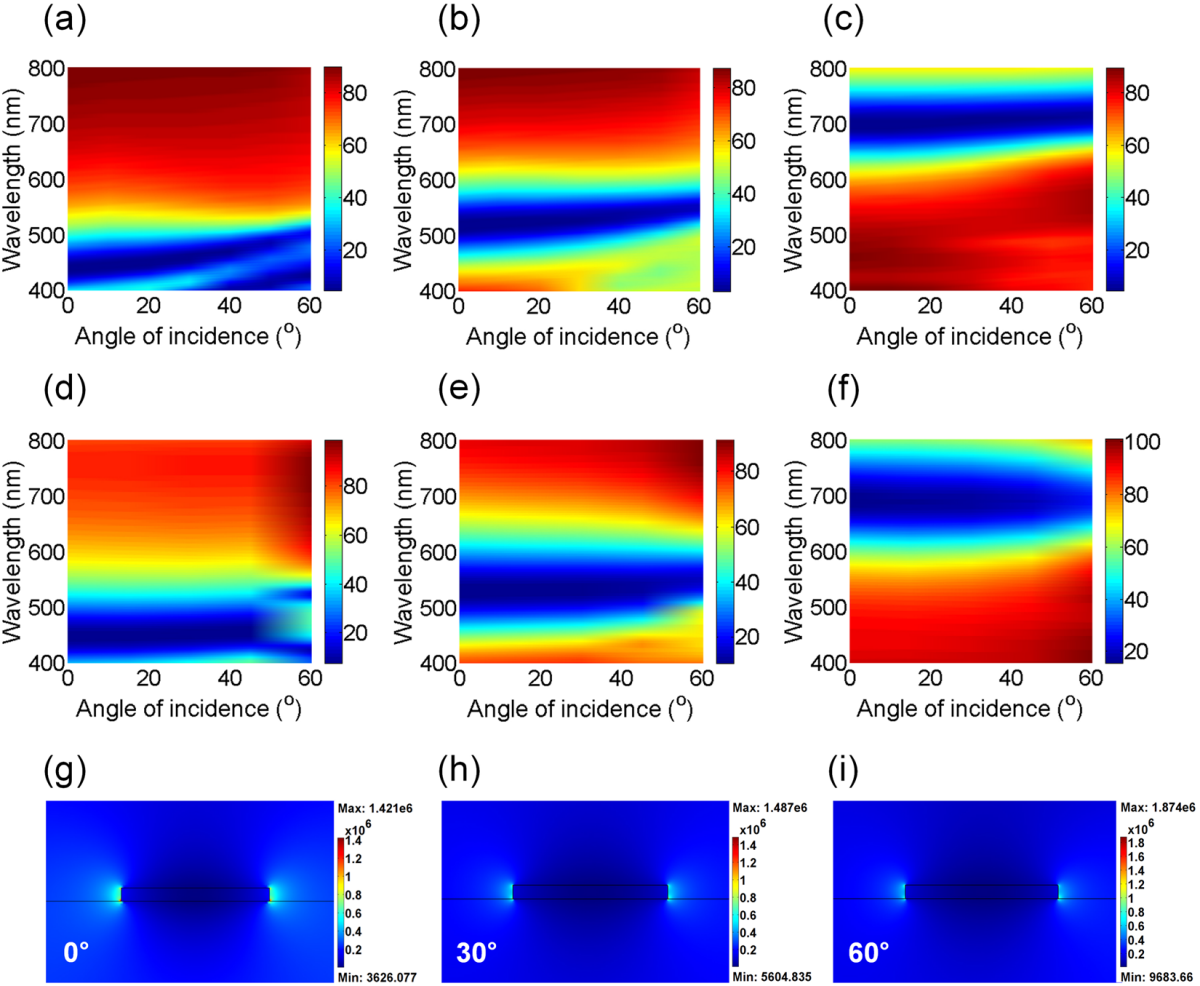
(**a**–**c**) Simulated and (**d**–**f**) measured angle-resolved transmission spectra of the ultrathin metallic nanowire-based structural color filters presenting the plasmonic resonances (i.e., dark blue color) are invariant with respect to angles of incidence up to 60°. Such angle-insensitive features are ascribed to the localized surface plasmon resonance. Normalized electric field distributions in the plasmonic color filters with 8 nm-thick Ag layer at (**g**) 0°, (**h**) 30° and (**i**) 60°. It is apparent that the electric field profiles show the localized surface plasmon resonance characteristics where the field decays within tens of nanometers at corners of the subwavelength Ag gratings regardless of the incident angles. Bright and dark colors represent high and low field intensity, respectively.

**Figure 5:**
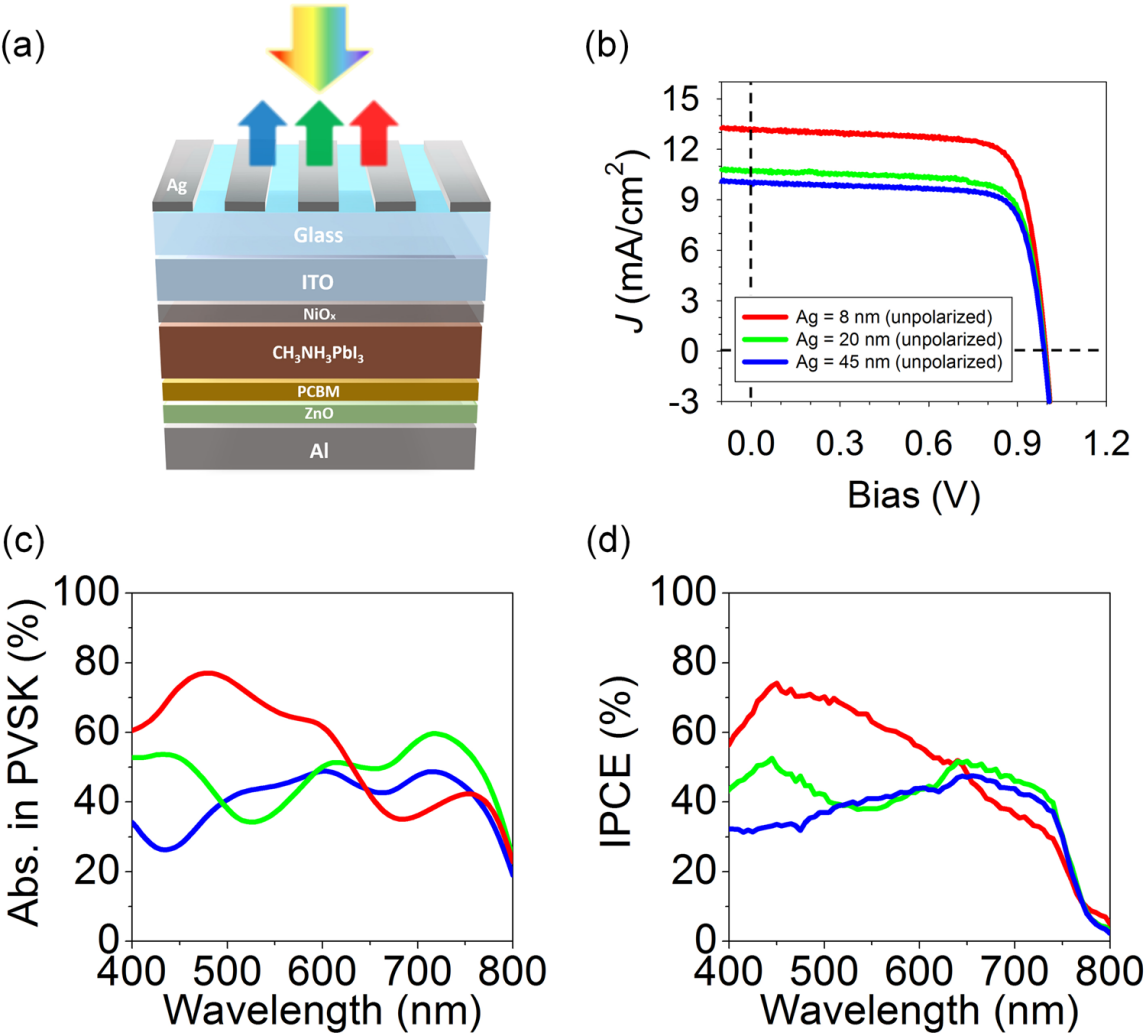
(**a**) Schematic illustration describing how perovskite (PVSK) solar cells, composed of ITO/NiO_x_/CH_3_NH_3_PbI_3_/PCBM/ZnO/Al, harvest transmitted light through the plasmonic color filters. (**b**) Current density (*J*)-voltage (*V*) characteristics of the perovskite solar cells integrated with the RGB plasmonic reflective color filters. (**c**) Simulated absorption spectra in a PVSK after passing through the RGB plasmonic color filters un﻿der ﻿unpolarized ﻿lig﻿ht illuminatio﻿n condition. (**d**) Measured incident photon-to-current efficiency (IPCE) spectra under ﻿unpolarized lig﻿ht illumination.

Since the LSPR is employed in this study, it is expected that the resulting reflection/transmission spectra are insensitive with respect to the angle of incidence. Figure 4(a)–(c) present 2D contour plots of the simulated transmission as a function of wavelength and incident angles for TM polarization. Positions of the blue color in 2D plots that represent the LSPR (i.e., transmission valley) remain constant in wavelength over a wide range of angles of incidence up to 60°, showing flat dispersion characteristics and hence angle-insensitive performance. The simulation results are in good agreement with experimental angle-resolved transmission spectra measured by using the spectrometer (V-770 UV-Visible-Near Infrared Spectrophotometer, JASCO) as presented in Fig. 4(d)–(f). As the spectral response of the plasmonic color filters does not noticeably change at oblique angles of incidence, the color purity is nearly maintained with increasing the angles of incidence as shown in Figure S6. Figure 4(g)–(i) describe cross-sectional distributions of normalized electric field into the plasmonic color filters comprising 8 nm-thick Ag subwavelength gratings at several oblique angles of incidence under the TM-polarized illumination, displaying that the nanostructures have the LSPR modes with a decay length of a few tens of nanometers at each corner of the subwavelength metallic gratings. Such strong electric fields, only confined at the corners of the nanogratings regardless of the angles of incidence, are also observed in the devices with 20 and 45 nm-thick Ag gratings, as depicted in Figure S7. In contrast to TM polarization, a strong field enhancement at the corners of the subwavelength gratings is not observed for TE polarization as shown in Figure S8. Blue and red denote low and high intensity of the electric field, respectively.  Lastly, we investigated the conceptual devices for a power-generating colored solar panel by integrating the plasmonic color filters with a planar heterojunction PVSK solar cell structure (ITO/NiO_x_/CH_3_NH_3_PbI_3_/PCBM/ZnO/Al)^42^, which can harvest the transmitted light through the plasmonic color filters as illustrated in Fig. 5(a). As shown in the schematic diagram, light is incident upon the plasmonic color filters for color generation first and then reaches the solar cells underneath the color filters to generate electricity. The colors and the reflection spectra of the plasmonic filter-integrated PVSK solar cells remain almost the same as the filter-only samples shown in Fig. 2(e) regardless of the thickness of underlying PVSK layer, and therefore the PVSK solar cell structures were optimized independently. Figure 5(b) shows current density (*J*) and voltage (*V*) characteristics of the PVSK solar cells after passing through the plasmonic RGB reflective color filters. Different spectral responses of the plasmonic reflective color filters lead to different values of *J*_sc_ (PCE): 13.17 (10.12), 10.77 (8.17) and 10.00 (7.72) mA/cm^2^ (%) for the RGB colored solar cells, respectively, while there is not much change in both *V*_oc_ and FF in all the devices. The electrical performances of the fabricated devices are summarized in Table 1 and Table S1. *J*_sc_ and the corresponding PCE of the PVSK solar cell after passing through the red color filter are the highest among the three cells, which is attributed to the fact that creating the resonance at longer wavelengths (i.e., red color) allows the shorter wavelengths of visible light, where the PVSK semiconductor has a high absorption coefficient, to be efficiently harvested by the solar cell without any disturbance. In contrast, the PVSK solar cell integrated with the blue color filter shows the lowest *J*_sc_ and PCE, as a certain portion of the shorter wavelengths needs to be utilized for the color generation, which cannot be scavenged by the solar cell. Such phenomena can also be observed by exploring the calculated optical absorption in a photoactive layer of the solar cells and the measured incident photon-to-current efficiency (IPCE). Figure 5(c) presents simulated optical absorption spectra in a PVSK layer under the unpolarized light illumination, which show good agreement with experimentally measured IPCE as depicted in Fig. 5(d). Calculated *J*_sc_ values from the simulated absorption profiles in the PVSK photoactive layer (measured IPCE spectra) are found to be 12.74 (13.05), 10.73 (10.71) and 9.40 (9.71) mA/cm^2^ for the RGB colored solar cells (Figure S9), respectively, all of which match well with *J*_sc_ values from *J*-*V* characteristics in Table 1 and Table S1. Simulated absorption spectra in the PVSK photoactive layer and measured IPCE profiles for both TE and TM polarizations are provided in Figure S10. The *J*-*V* curve and the IPCE of an optimized PVSK solar cell without the plasmonic color filter are given in Figure S11 for reference. The average and best values of device performances under both unpolarized and polarized (TM and TE) light conditions are summarized in Table S1. All *J*-*V* characteristics of our PVSK solar cells showed negligible hysteresis depending on scan directions, indicating stable operation of each cell (Figures S11a and S12).

**Table 1:** Summary of *V*_oc_, *J*_*sc*_,﻿ FF and PCE of the best performing PVSK solar cells integrated with the plasmonic color filters.

	*V*_oc_ (V)	*J*_sc_ (mA/cm^2^)	FF	PCE (%)
Red	1.00	13.17	0.77	10.12
Green	0.99	10.77	0.77	8.17
Blue	0.99	10.00	0.78	7.72

*Under AM 1.5 G 100 mW·cm^−2^ illumination condition.

In order to further improve the efficiency of the colored perovskite solar cells, the color filters that only consist of optically transparent dielectric materials without any lossy media such as metals can be incorporated with the perovskite solar cells. Moreover, any types of solar cells, which harvest near-infrared region of the solar spectrum and exhibit transparency in the visible wavelength range, can be integrated with the colored perosvskite solar cells to provide additional absolute efficiency.

## Conclusion

In summary, we have presented angle-insensitive color-generating PVSK solar cells, integrated with the plasmonic color filters comprising the ultrathin single metallic layer patterned at the subwavelength scale on the transparent substrate, for highly efficient and easily tunable decorative power-generating panels. The LSPR in the ultrathin nano-strip resonators was employed to produce the reflective RGB colors that are insensitive to the incident angles up to 60°, and simultaneously the complementary spectra in the visible wavelength range with negligible absorptions can be transmitted through the transparent substrate and therefore efficiently harvested by the PVSK solar cells underneath the plasmonic color filters, generating 10.12% (red), 8.17% (green) and 7.72% (blue) of PCEs. The presented approach could open important possibilities toward self-powered devices, decorative solar panels, energy-saving reflective displays and BIPV.

## Methods

### Plasmonic color filter fabrication

The large-area Ag nanogratings were fabricated by nanoimprint lithography (NIL)-based processes. NIL was performed in an EITRE-8 naonoimprinter (OBDUCAT) using a silicon dioxide (SiO_2_) mold on a poly(methyl methacrylate) (PMMA) resist spin-cast on the glass substrates, at a pressure of 35 bar and a temperature of 140 °C, for 5 min. After cooling and demolding, chromium (Cr) was selectively deposited on each sidewall of the imprinted grating structures by angled deposition. The Cr deposited on the resist patterns induced the undercut structures during O_2_ reactive ion etching (RIE), facilitating the lift-off process. O_2_ RIE (10 sccm O_2_, 40 mTorr chamber pressure, and 40W bias power), deposition of Cu (1 nm) and Ag using the e-beam evaporator, and the lift-off process completed the fabrication of nanograting structures on a substrate. 1 nm-thick Cu was a seed layer to improve the uniformity of the following Ag layer^43^.

### Perovskite solar cell fabrication

Acetone, isopropyl alcohol (IPA), and deionized (DI) water were sequentially used to clean the patterned ITO-coated glass substrates via ultrasonication. The ITO substrates were treated with oxygen plasma for 5 min before use to improve the wettability of following solution. NiO_x_ nanoparticle (NP) solution was spin-cast on the ITO layer and annealed at 150 °C for 10 min. After annealing, the sample was transferred to Nitrogen (N_2_) box. To synthesize NiO_x_ NPs, Ni(NO_3_)_2_·6H_2_O (0.025 mol) was dissolved in 5 ml DI water, and then stirred for 10 min. After that, a polyethylene glycol (PEG) solution, in which 1.5 g PEG was dissolved in 5 ml DI water, was dropped into Ni(NO_3_)_2_·6H_2_O solution drop by drop, and then the solution was stirred for 15 min. The pH of the solution was adjusted to 10 by further adding a NaOH solution, in which 0.05 mol NaOH was dissolved in 5 ml DI water, and it was stirred for 15 min. After filtering the solution, the precipitation was dried at 80 °C for 8 h, and then annealed at 285 °C for 120 min in furnace. NiO_x_ NPs were dispersed to DI water to obtain NiO_x_ NP solution. CH_3_NH_3_PbI_3_ perovskite solution was prepared by dissolving PbI_2_ (Sigma Aldrich) and CH_3_NH_3_I (1:1 molar ratio) in the solvent mixture of γ-butyrolactone (GBL) and dimethylsulfoxide (DMSO) (7:3 v/v) for a total concentration of 1.1 M in a N_2_ atmosphere. The solution was stirred at 70 °C for at least 12 h before being used. The perovskite layer was formed onto the NiO_x_ hole transport layer by a consecutive two-step spin-casting process at 1,000 and 5,000 rpm for 10 and 20 s, respectively. During the second spin-casting step, the substrate was treated with toluene drop-casting. The substrate was dried on a hot plate at 100 °C for 10 min. PCBM (20 mg/ml in chlorobenzene) was then spin-cast on top of the perovskite layer and ZnO layer was added by spin-casting ZnO NP solution (Sigma Aldrich) on PCBM as an electron transport layer (ETL). Those perovskite photoabsorber, PCBM and ZnO layers were prepared in a N_2_ box. Finally, the samples were transferred into a thermal evaporator and Al (80 nm) was thermally deposited at a base pressure of 4 × 10^−6^ torr. The active area of the solar cell was about 0.06 cm^2^.

### Simulation and characterization

1D plasmonic nanograting patterns were created on a glass substrate, while multilayer thin film PVSK solar cells were placed on the opposite side of the glass substrate in the optical simulation. A built-in mesh parameter set in COMSOL Multiphysics (Fine) was utilized to mesh the entire device structure, whereas Normal was used for the incident medium. Floquet periodic boundary conditions were used for surfaces of a substrate and incident medium, while port boundary conditions were used for a top of the incident medium and a bottom of the substrate. The type of the light illumination source is TE, TM and unpolarized light. Incident light with TM polarization was illuminated from the plasmonic subwavelength grating side, where some portion of incident light was reflected with insignificant absorptions by the LSPR for reflective color generation, and the complementary spectrum of the visible light was transmitted toward the PVSK solar cell at the other side for electricity generation. The simulated transmission and reflection spectra for TE and unpolarized light were also performed. Spectral reflectance and transmittance curves, angle-resolved reflection and transmission spectra, normalized electric field distributions and optical absorption profiles in a perovskite photoactive layer were calculated by using a commercial simulation software, COMSOL Multiphysics, which was based on finite element method. The *J*_sc_ values from both simulation and experiment were calculated by using the following equation:

1 $${J}_{SC}={\int }_{400nm}^{800nm}\frac{e\lambda }{hc}QE(\lambda ){I}_{AM1.5}(\lambda )d\lambda $$

where *e*, *λ*, *h*, *c* and I_AM1.5_(*λ*) are elementary charge, wavelength, Plank constant, speed of light and AM 1.5 solar spectral irradiance, respectively, under the assumption that the quantum efficiency is equal to the absorption characteristics in the PVSK photoactive layer (i.e., internal quantum efficiency is 100%). The refractive index of Ag, measured by using a spectroscopic ellipsometer (Elli-SE, Ellipso Technology Co.), and 1.46 for glass substrates were used in the simulation. The reflection spectra were characterized by using a spectrometer (Elli-RSc, Ellipso Technology Co.), and the angle-resolved transmission spectra were measured by using a spectrometer (V-770 UV-Visible-Near Infrared Spectrophotometer, JASCO). A solar simulator (PEC-L01, Peccell Technologies, Inc.) with an AM 1.5 G filter was used to provide 100 mW·cm^−2^ of illumination on the PV cells with the intensity calibrated using a Si photodiode, equipped with an infrared cut-off filter (KG5) to reduce spectral mismatch, and the active area of PV cell was defined using an opaque metal mask (2 mm × 3 mm) to reduce the influence of the scattered light. *J*-*V* characteristics were obtained using an Ivium technology Ivium compactstat by scanning the *J*-*V* curves at a 0.05 V·s^−1^ scan rate. The incident-photon-to-electron conversion efficiency (IPCE) was measured under short-circuit conditions using ABET Technology 10500 solar simulator as the light source and SPECTRO Mmac-200 as the light solution.

## Electronic supplementary material

Supplementary information
