# Supplementary material for: Highly Efficient Colored Perovskite Solar Cells Integrated with Ultrathin Subwavelength Plasmonic Nanoresonators
Source: Sci Rep. 2017 Sep 6;7:10640. doi: 10.1038/s41598-017-10937-3 (PMC5587539; doi:10.1038/s41598-017-10937-3)
Supplement: Supplementary file 1 — Supplementary information [file 41598_2017_10937_MOESM1_ESM.pdf]

Supplementary Information

Highly Efficient Colored Perovskite Solar Cells Integrated with Ultrathin Subwavelength Plasmonic Nanoresonators

Kyu-Tae Lee[1,*], Ji-Yun Jang[2,*], Jing Zhang[2], Sung-Mo Yang[2], Sanghyuk Park[3] & Hui Joon Park[2,4]

Corresponding Author

Prof. Hui Joon Park

E-mail: huijoon@ajou.ac.kr / Tel: +82-31-219-2577 / Fax: +82-31-219-2208

[1]Department of Materials Science and Engineering, University of Illinois at Urbana-Champaign, Urbana, Illinois 61801, United States

[2]Department of Energy Systems Research, Ajou University, Suwon 16499, Korea

[3]Department of Chemistry, Kongju National University, Chungnam, 32588, Korea

[4]Department of Electrical and Computer Engineering, Ajou University, Suwon 16499, Korea

**These authors contributed equally to this work*

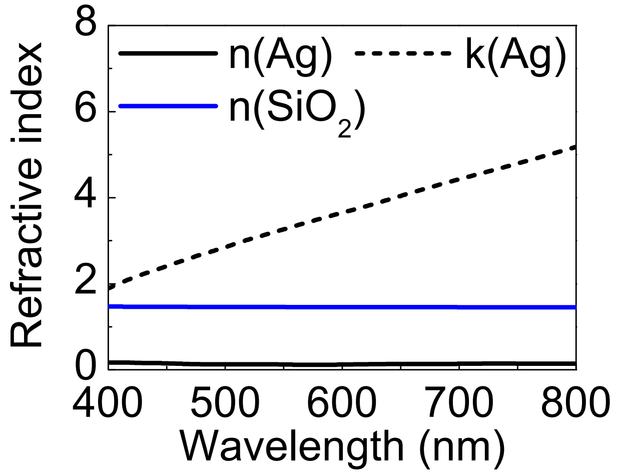

Figure S1. Refractive index of Ag measured by a spectroscopic ellipsometer (Elli-SE, Ellipso Technology Co.) and 1.46 for a glass substrate.

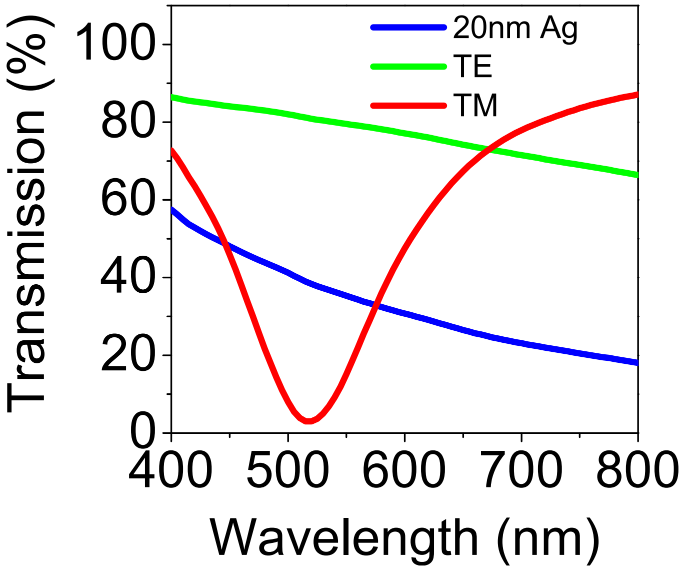

Figure S2. Simulated transmission spectra of a bare 20 nm-thick Ag film (blue) and patterned Ag gratings (t= 20nm, P=220 nm, W=90 nm) for TE (green) and TM (red) polarizations.

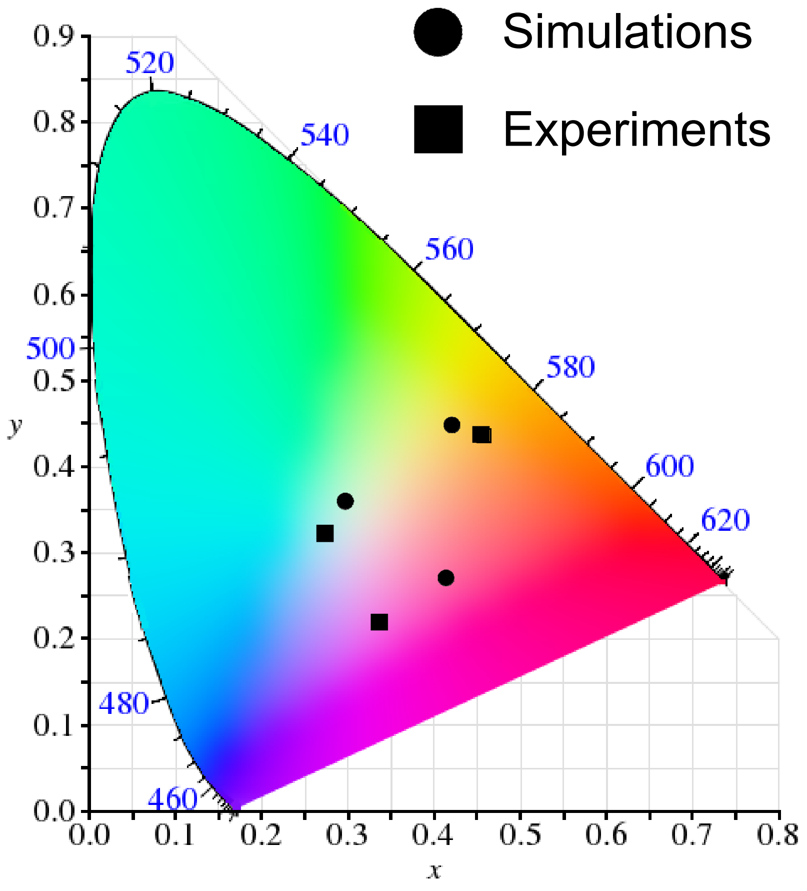

Figure S3. Chromaticity diagram of simulated (circles) and measured (squares) transmission spectra for TM polarization.

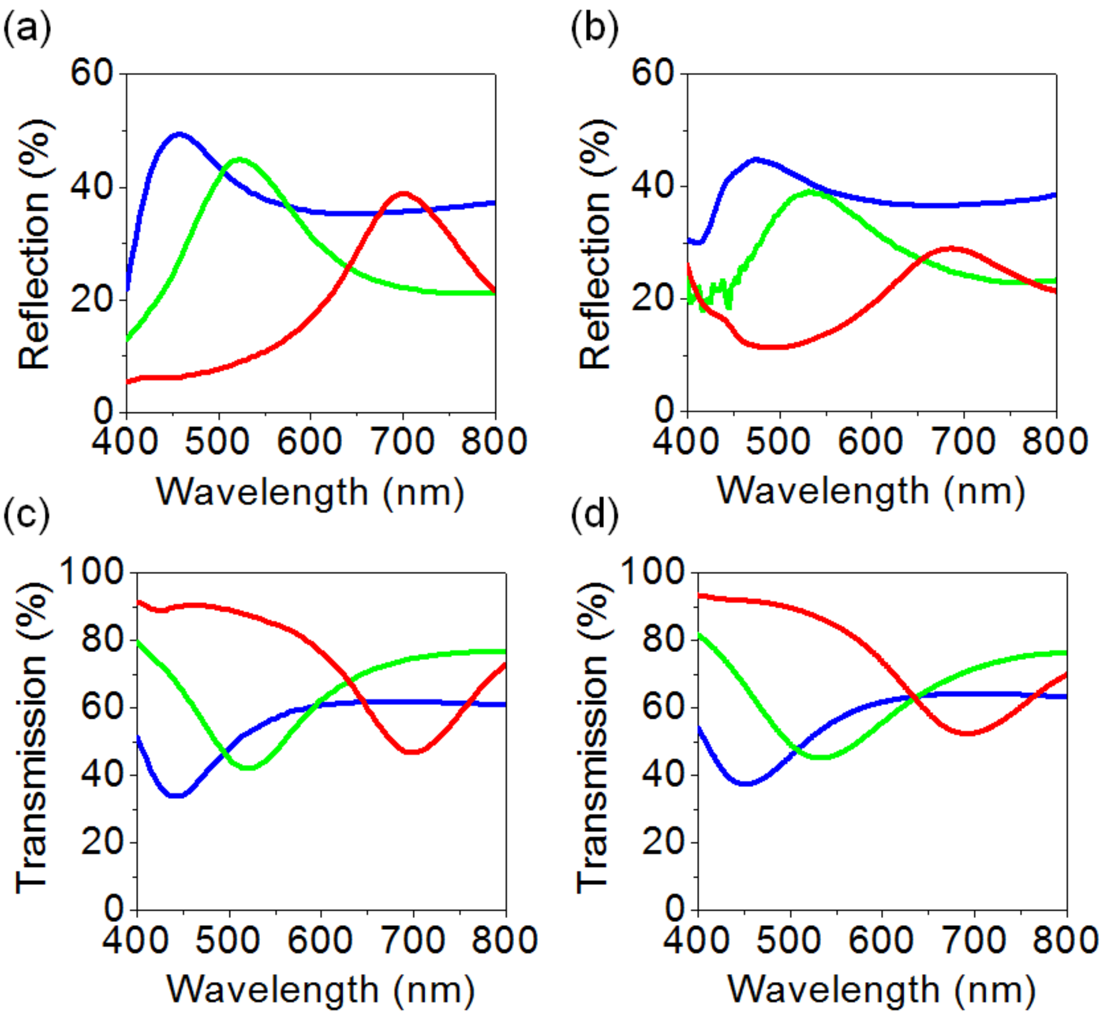

Figure S4. (a), (c) Simulated and (b), (d) measured reflection and transmission spectra of the plasmonic color filters for unpolarized normal incident light.

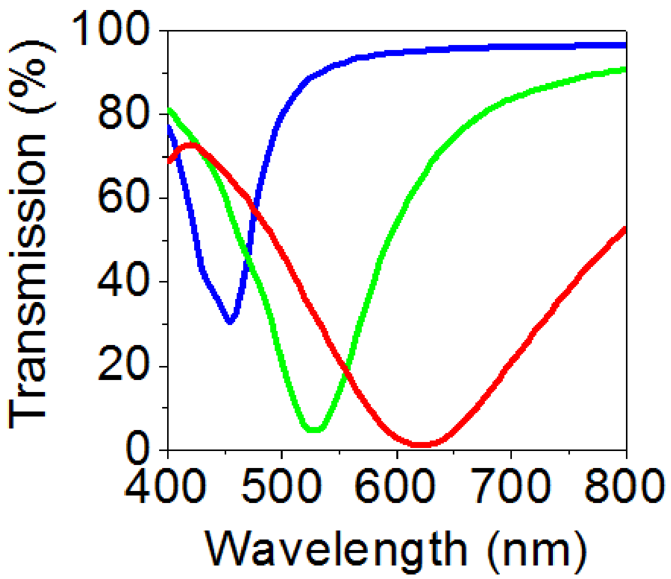

Figure S5. Simulated transmission spectra of the ultrathin nanowire-based plasmonic color filters at fixed thickness for creating individual colors in pixel unit via one-step fabrication for the TM normal incident light (Yellow: P= 280 nm, W= 50 nm, t=20 nm, Magenta: P= 280 nm, W= 90 nm, t=20 nm, Cyan: P= 280 nm, W= 140 nm, t=20 nm).

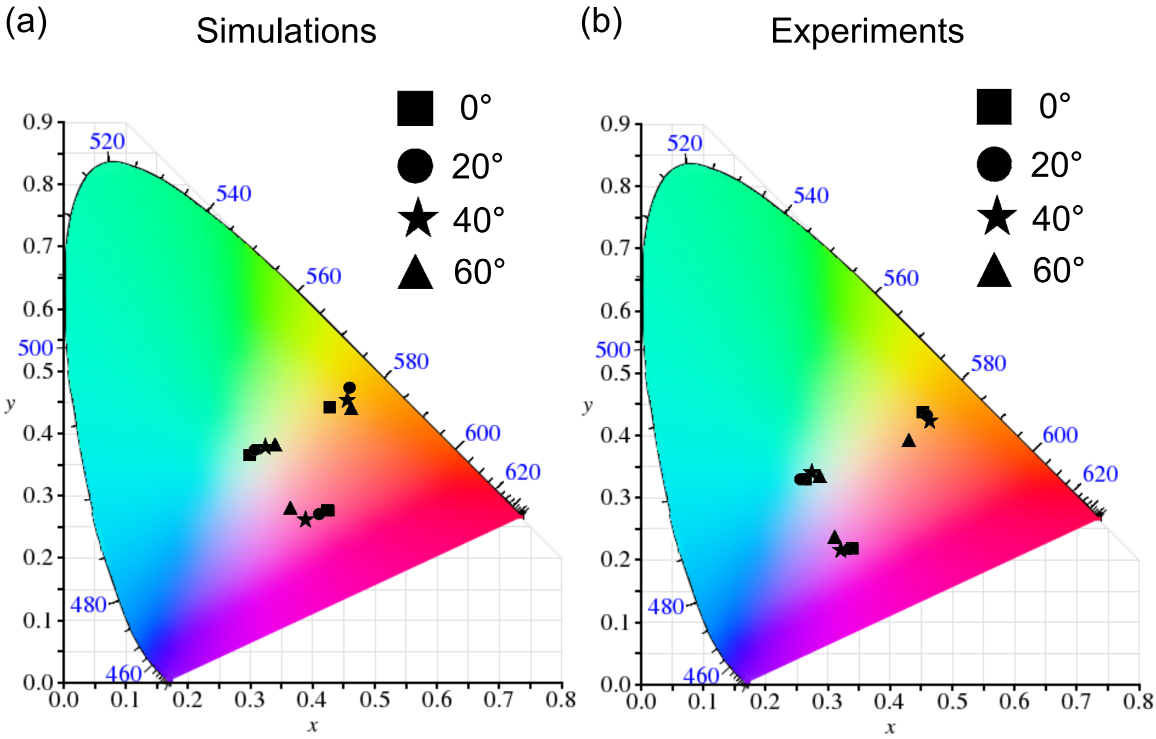

Figure S6. Chromaticity diagram of (a) simulated and (b) measured transmission spectra at several oblique angles of incidence.

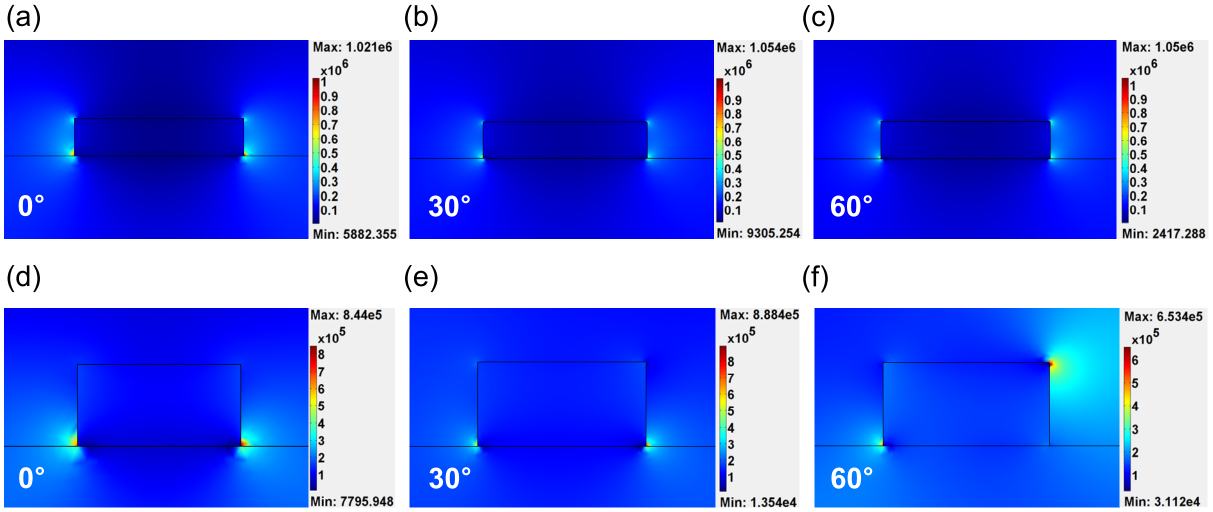

Figure S7. Normalized electric field distributions in the plasmonic color filters with (a) - (c) 20 nm-thick and (d) - (f) 45 nm-thick Ag layer at 0°, 30° and 60° for TM polarization.

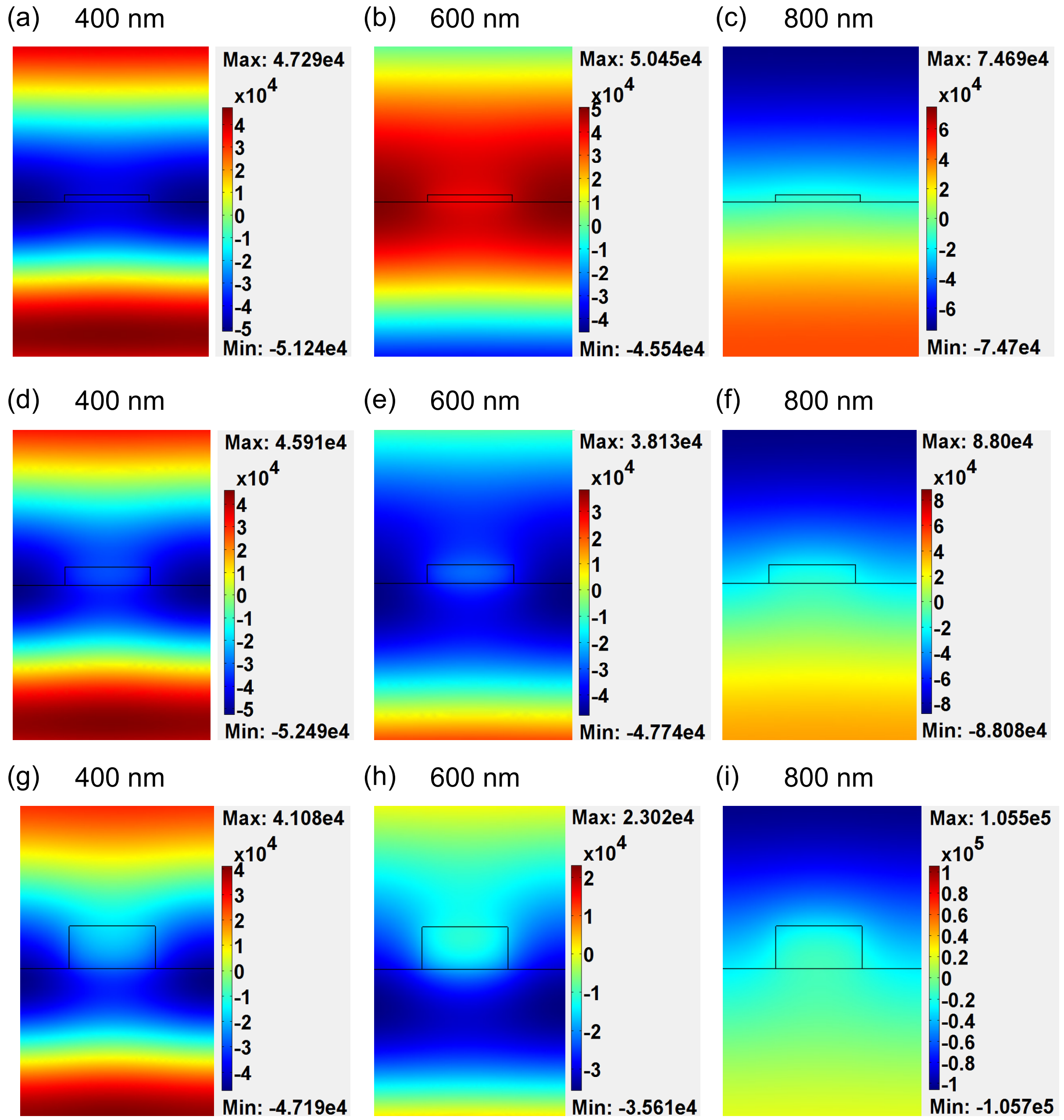

Figure S8. Normalized electric field distributions in the plasmonic color filters with (a) - (c) 8 nm-thick, (d) - (f) 20 nm-thick and (g) - (i) 45 nn-thick Ag layer at 0° for TE polarization.

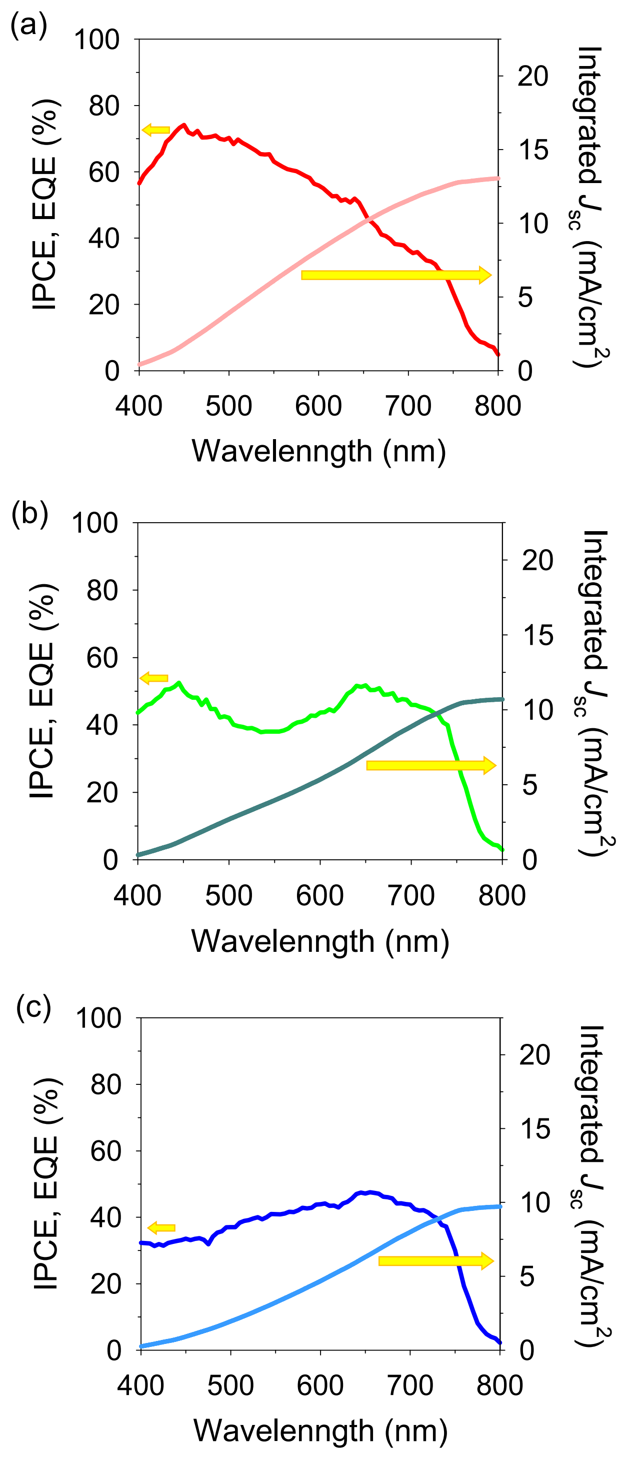

Figure S9. Measured external quantum efficiency (EQE) spectra and integrated photocurrent density J_{sc} of (a) red colored PV, (b) green colored PV and (c) blue colored PV.

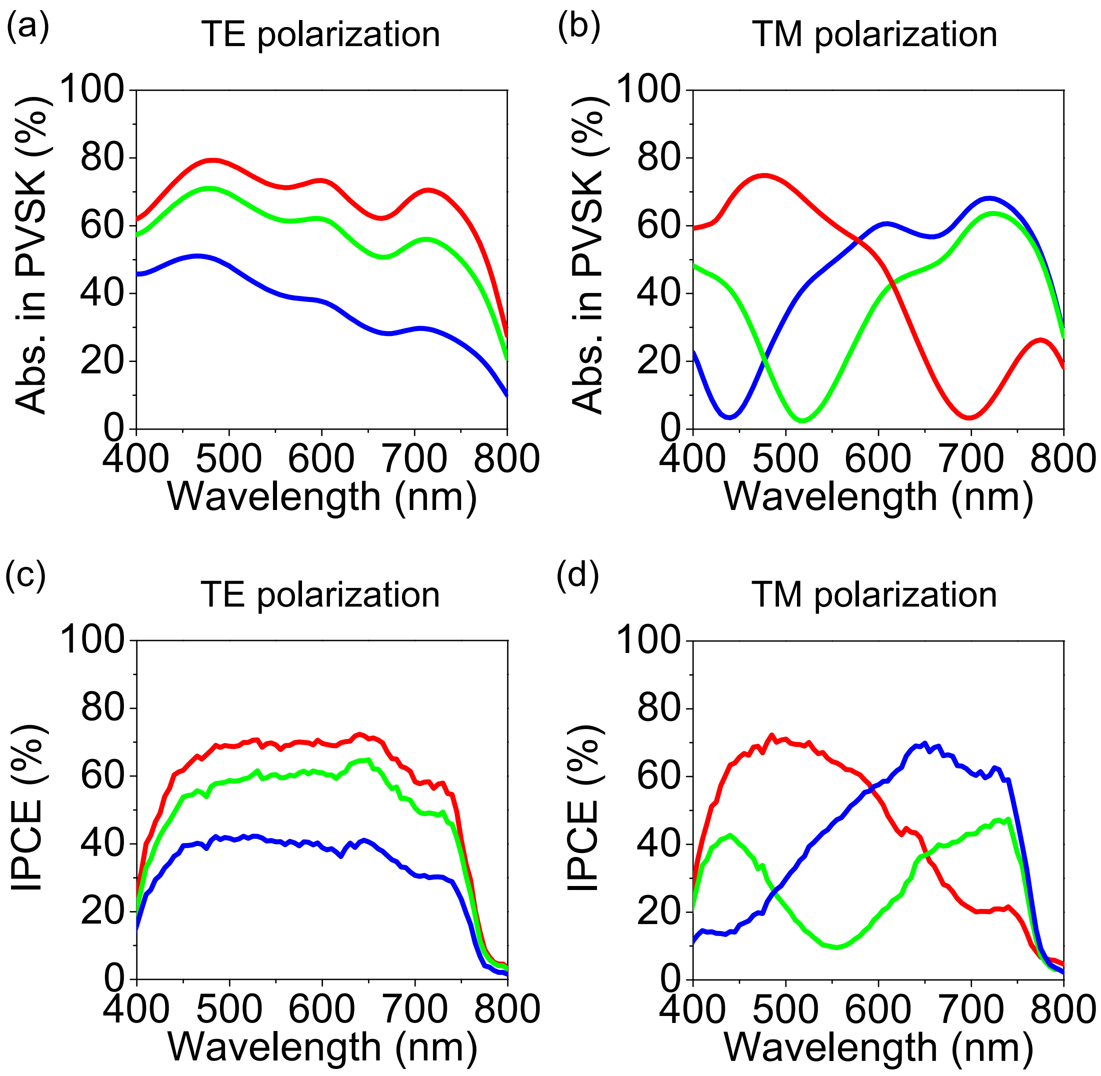

Figure S10. (a) - (b) Simulated absorption spectra in a perovskite layer after passing through the RGB plasmonic color filters for TE and TM polarizations. (c) - (d) Measured incident photon-to-current efficiency (IPCE) spectra for TE and TM polarizations.

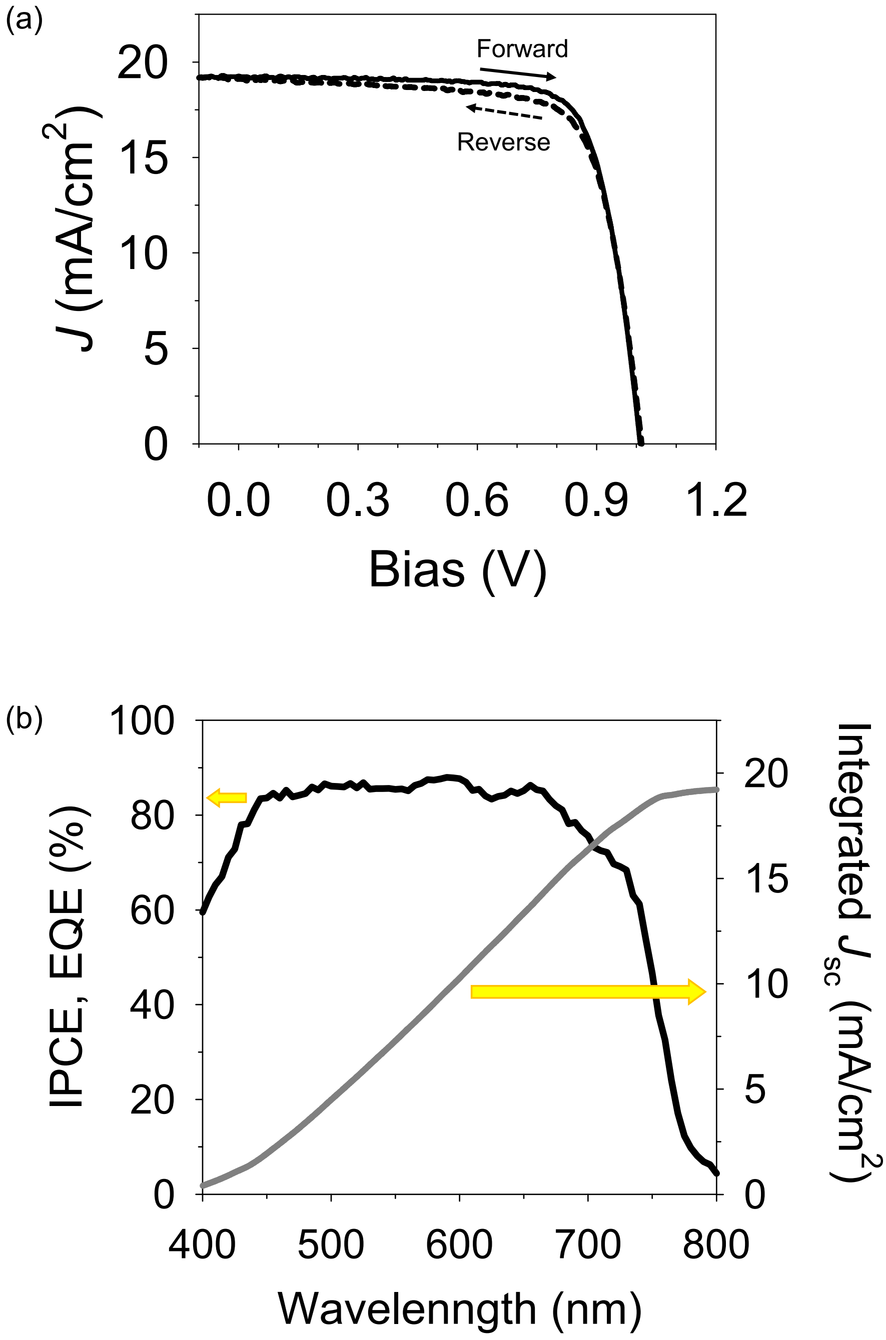

Figure S11. (a) *J*-*V* curve of the PVSK solar cell device without the plasmonic color filter as a control (Under AM 1.5G 100 mW·cm^{-2} illumination condition). (b) External quantum efficiency (EQE) of the PVSK solar cell device without the plasmonic color filter.

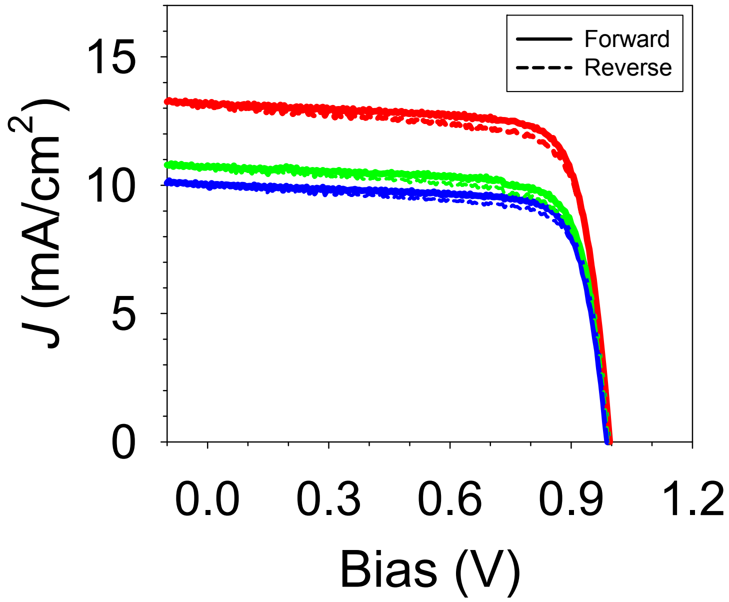

Figure S12. *J-V* curves of colored solar cells scanned in forward and reverse directions. The solid and dashed lines are data scanned in forward and reverse directions, respectively. All data were measured at AM 1.5G with an intensity of 100 mW·cm^{-2}.

Table S1. Detailed summary of J_{sc}, V_{oc}, FF and PCE of the PVSK solar cells

		V_{oc} (V)	J_{sc} (mA/cm^2)	FF	PCE (%)
Without CF	Unpolar	1.04 (1.02)	19.07 (19.67)	0.72 (0.77)	14.22 (15.51)
Red	Unpolar	1.00 (1.00)	12.61 (13.17)	0.77 (0.77)	9.73 (10.12)
	TM	1.00 (1.02)	5.71 (7.88)	0.72 (0.75)	8.25 (11.96)
	TE	1.01 (1.02)	7.63 (7.68)	0.72 (0.74)	11.03 (11.51)
Green	Unpolar	0.99 (0.99)	9.94 (10.77)	0.77 (0.77)	7.59 (8.17)
	TM	0.98 (0.98)	3.18 (3.58)	0.75 (0.75)	4.64 (5.22)
	TE	1.01 (1.01)	6.38 (6.58)	0.74 (0.73)	9.46 (9.69)
Blue	Unpolar	1.00 (0.99)	9.22 (10.00)	0.77 (0.78)	7.11 (7.72)
	TM	1.00 (1.00)	5.55 (7.14)	0.74 (0.77)	8.24 (10.92)
	TE	1.00 (1.00)	4.36 (5.61)	0.74 (0.77)	6.47 (8.58)

Numbers are average values of 20 devices for each condition (values in parentheses are from the best performing devices).

Unpolarization: AM 1.5G with 100 mW·cm^{-2} intensity

TM and TE polarization: AM 1.5G with 50 mW·cm^{-2} intensity (under polarizer)
